# Association of graph-based spatial features with overall survival status of glioblastoma patients

**DOI:** 10.1038/s41598-023-44353-7

**Published:** 2023-10-09

**Authors:** Joonsang Lee, Shivali Narang, Juan Martinez, Ganesh Rao, Arvind Rao

**Affiliations:** 1 Department of Computational Medicine and Bioinformatics, University of Michigan, Ann Arbor, MI 48109 USA; 2 Department of Bioinformatics and Computational Biology, The University of Texas MD Anderson Cancer Center, Houston, TX USA; 3 Department of Neurosurgery, The University of Texas MD Anderson Cancer Center, Houston, TX USA

**Keywords:** Biomarkers, Cancer imaging

## Abstract

Glioblastoma is the most common malignant brain tumor with less than 15 months median survival. To aid prognosis, there is a need for decision tools that leverage diagnostic modalities such as MRI to inform survival. In this study, we examine higher-order spatial proximity characteristics from habitats and propose two graph-based methods (minimum spanning tree and graph run-length matrix) to characterize spatial heterogeneity over tumor MRI-derived intensity habitats and assess their relationships with overall survival as well as the immune signature status of patients with glioblastoma. A data set of 74 patients was studied based on the availability of post-contrast T1-weighted and T2-weighted fluid attenuated inversion recovery (FLAIR) image data in The Cancer Image Archive (TCIA). We assessed the predictive value of MST- and GRLM-derived features from 2D images for prediction of 12-month survival status and immune signature status of patients with glioblastoma via a receiver operating characteristic curve analysis. For 12-month survival prediction using MST-based method, sensitivity and specificity were 0.82 and 0.79 respectively. For GRLM-based method, sensitivity and specificity were 0.73 and 0.77 respectively. For immune status, sensitivity and specificity were 0.91 and 0.69, respectively, for the GRLM-based method with an immune effector. Our results show that the proposed MST- and GRLM-derived features are predictive of 12-month survival status as well as the immune signature status of patients with glioblastoma. To our knowledge, this is the first application of MST- and GRLM-based proximity analyses for the study of radiologically-defined tumor habitats in glioblastoma.

## Introduction

Glioblastoma is a common primary brain tumor known for its aggressive malignant behavior. Prognosis for patients with glioblastoma remains very poor with the median overall survival duration between 9 and 15 months despite multimodality treatments such as surgical resection followed by combination of radiation therapy and chemotherapy (temozolomide)^1–3^. Several studies have been proposed to improve the diagnostic performance of magnetic resonance imaging (MRI) for cancer using various techniques such as computer-based image analyses^4,5^, imaging features analysis^6–8^, machine learning techniques^9^, imaging-genomics analysis^10,11^.

From MRI analysis studies of glioblastoma patients, it has been suggested that intensity-level heterogeneity within the tumor is indicative of multiple tumor regions with distinct MRI intensity characteristics that might respond differently to treatment regimens^12^. This has implications for the assessment of patient prognosis in glioblastoma^13,14^. More specifically, the presence of intensity-level heterogeneity within the tumor indicates the existence of different regions within the tumor, each exhibiting distinct MRI intensity characteristics. Understanding the heterogeneity allows oncologists to tailor treatment plans based on the specific characteristics of different tumor regions. This could lead to more targeted and effective therapies for each patient. Also, different tumor regions may have varying aggressiveness and growth rates. Analyzing heterogeneity allows for a more accurate assessment of the tumor’s behavior and potential progression. This helps in providing patients with more precise prognostic information. Based on multiparametric measurements of different MRI sequences, these habitats characterize regional variations in blood flow, cell density, and necrosis^12^. Apart from the abundance of these habitats, the spatial extents and proximity of these habitats have physiologic and clinical relevance for assessment of treatment response^12,15–17^.

In this study, we identified four distinct groups of voxel intensities (habitats) within the tumor ROI across different MR sequences and characterized the spatial relationships of these derived habitats with graph-based methods such as a minimum spanning tree (MST) construction and graph run-length matrices (GRLM). In previous breast cancer studies, graph (MST-derived) features have been used to understand the proximity relationships between distinct immunohistochemical entities (cell types) within hematoxylin and eosin (H&E) pathology slides^18^. These MST-based features successfully distinguished samples of high and low lymphocytic infiltration extent with a classification accuracy greater than 90% in breast cancer^18^. Based on the success of this approach, we hypothesized that characterizing the spatial relationship between radiologically-distinct habitats of a tumor using MST or GRLM approaches might have predictive value for underlying clinical outcome in glioblastoma.

Another graph-based characterization called GRLM^19^ was also used to characterize the spatial heterogeneity of a tumor to predict clinical outcome in glioblastoma, based on the idea of gray-level run-length matrices. The gray-level run-length method is one of popular methods extracting high order statistical features in texture analysis^20^. In this study, we used GRLM to compute the runs of radiologically defined tumor habitats on a region of interest (ROI) image to obtain a run-length matrix instead of counting runs of voxel intensities. In addition, Researchers have been increasingly studying the role of the immune system in the development, progression, and potential treatment of glioblastoma. The immune microenvironment within the tumor, which includes various immune cells and signaling molecules, plays a critical role in shaping the tumor’s behavior and response to treatment. In this study, the immune gene signature status was determined for each patient using single-sample gene set enrichment analysis (ssGSEA), based on the established immune effector (IE) and immune suppressor (IS) responses^21–25^. The ssGSEA process assigned an enrichment score to measure how genes within a specific gene set are collectively upregulated or downregulated within a sample.

The purpose of this study is to evaluate the prognostic significance of higher-order spatial proximity characteristics from habitats using quantitative metrics derived from graph-based methods. We aim to investigate the association of MST and GRLM-derived spatial proximity features of these tumor habitats with overall survival of glioblastoma patients as well as predicting immune signature status. In the broader context, this study aims to investigate relationships between imaging and phenotypic characterizations of the tumor, thereby augmenting the foundation for population-based correlation studies in glioblastoma.

## Materials and methods

### Data

A data set of 74 patients was studied based on the availability of post-contrast T1-weighted and T2-weighted fluid attenuated inversion recovery (FLAIR) image data in The Cancer Genome Atlas (TCGA), The Cancer Imaging Archive (TCIA—http://www.cancerimagingarchive.net/). The dataset consisted of 25 female and 49 male patients with de-novo (primary) glioblastoma, all of whom were classified as IDH-wild type astrocytoma. The patient demographics are summarized in Table 1. The mRNA expression data and clinical data such as survival information for these cases^26^ were obtained from the cBioPortal for Cancer Genomics (http://www.cbioportal.org). In this study, the previously defined immune effector and immune suppressor response^21^ were used to derive the immune gene signature status using single-sample gene set enrichment analysis: ssGSEA for each patient^22–25^. Basically, the IE response activates immune cells to eliminate threats like pathogens and abnormal cells. Immune cells like T cells, B cells, and natural killer cells are recruited to target and remove harmful entities. Conversely, the IS response moderates immune reactions to prevent excessive active activity, avoiding tissue damage and chronic inflammation. Regulatory T cells and immune checkpoint molecules contribute to maintaining immune balance. The study employs ingle-sample gene set enrichment analysis (ssGSEA) to evaluate these responses in patients. This technique assesses how genes linked to these responses are regulated within patient samples, providing insights into their immune gene signature status.

**Table 1:** Patient demographics.

Characteristics	OS ≤ 12 month	OS > 12 month	Total
Male (n)	19	30	49
Female (n)	12	13	25
Age (years)	61.6 ± 15.3	54.5 ± 15.4	57.5 ± 15.7
Overall survival (months)	5.9 ± 3.0	24.8 ± 12.7	16.9 ± 13.6

The data for age and overall survival are means with standard deviations.

*OS* overall survival.

**Figure 1:**
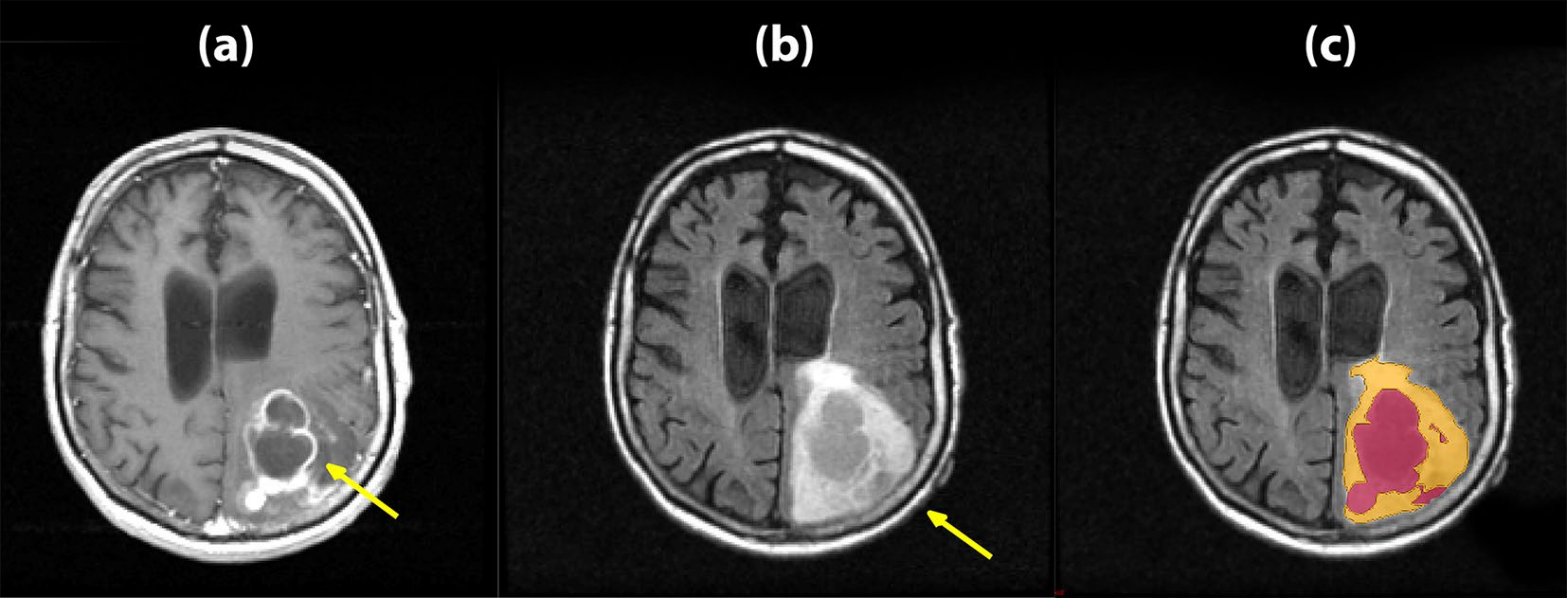
(**a**) T1 post-contrast image, (**b**) T2 FLAIR image with arrows point to the enhanced tumor areas, and (**c**) the segmentation results on T2 FLAIR image using MITK.

For predicting immune signature status, 34 patients were used based on availability among 74 patients. The MR images were preprocessed with registration, non-uniformity correction using N3^27^, voxel isotropic resampling, and intensity normalization^28^ before subsequent analysis. Registration of the T1 post-contrast image and T2 FLAIR image along with non-uniformity correction for MRI-artifacts were performed using the Medical Image Processing, Analysis, and Visualization (MIPAV) software^29^. The FLAIR MR image is registered to the T1 post-contrast image using affine transformation with 12 degrees of freedom along with trilinear interpolation. Segmentation was a semi-automated process for which MITK3M3 Image analysis toolkit was used. The clinicians used this tool to contour the tumor region on multiple slices with interpolation performed to obtain a 3D volumetric tumor mask. This step was performed independently on T1-post contrast as well as T2-FLAIR. In all the processes, readers were blinded to clinical/molecular characteristics. Voxel isotropic resampling was performed using the NIFTI toolbox in MATLAB to make voxel sizes isotropic (1 mm). The resulting T1 post-contrast image, T2 FLAIR image after preprocessing, and the segmentation results on T2 FLAIR image using MITK is shown in Fig. 1.

### Region of interest (ROI) delineation

A tumor ROI was segmented semi-automatically by two experts (radiologists) using the Medical Imaging Interaction Toolkit (MITK). The extent of tumor was defined using the contrast enhancing tumor region within the T1-post contrast image, and the areas of solid tumor, infiltrating tumor and edema regions within the T2-FLAIR image. The slice with the maximum tumor area in T1 post-contrast image and the corresponding slice from the T2 FLAIR image were selected for both MST and GRLM analysis.

### Radiologically defined tumor habitats

In this study, we used two different MR sequence images (T1 post-contrast image and T2 FLAIR image). For each MRI image-sequence, the voxels within the tumor ROIs were separated into low and high intensity group (habitat) using the two-component GMM. Four binary masks were prepared from these groups. A two-dimensional grid line was overlaid on each binary mask. The grid lines were equally spaced with a distance of 8 voxels (8 mm × 8 mm), chosen empirically. The voxel intensity values within the ROI for each patient were scaled to lie between zero and one by linear transformation, and then fitted using a two-component Gaussian mixture model (GMM)^30,31^. The threshold between two Gaussian groups was determined by calculating the average of the means of the two Gaussian populations underlying the low intensity voxel group and high intensity voxel group within the tumor, following a process similar to prior work^31^.

### Minimum spanning tree (MST) and graph run-length matrices (GRLM)

A spanning tree of a graph represents a tree that connects all the vertices. Each edge has an associated weight (or length), the weight of a tree represents the sum of all weights of its edges. Then, the MST can be defined as a spanning tree with the minimum weight of a tree among any other spanning trees.

The gray-level run-length matrix *M*(*i, j*) is a popular method in texture analysis that measures the variation of the voxel intensities to quantify intuitive qualities such as smoothness, coarseness, and roughness. Gray-level run-length can be defined as the number of runs with voxels of gray level *i* and run length *j* in a given direction. Basically, run-length matrix provides the coarseness of a texture in a specified direction. Runs of data represent sequences in which the same data value, gray level intensity, occurs in many consecutive voxel elements. In general, fine texture or high frequency tends to have more short runs with similar gray level intensities and coarse texture or low frequency tends to have longer runs of similar gray level intensities.

### Feature extraction with minimum spanning tree (MST)

**Figure 2:**
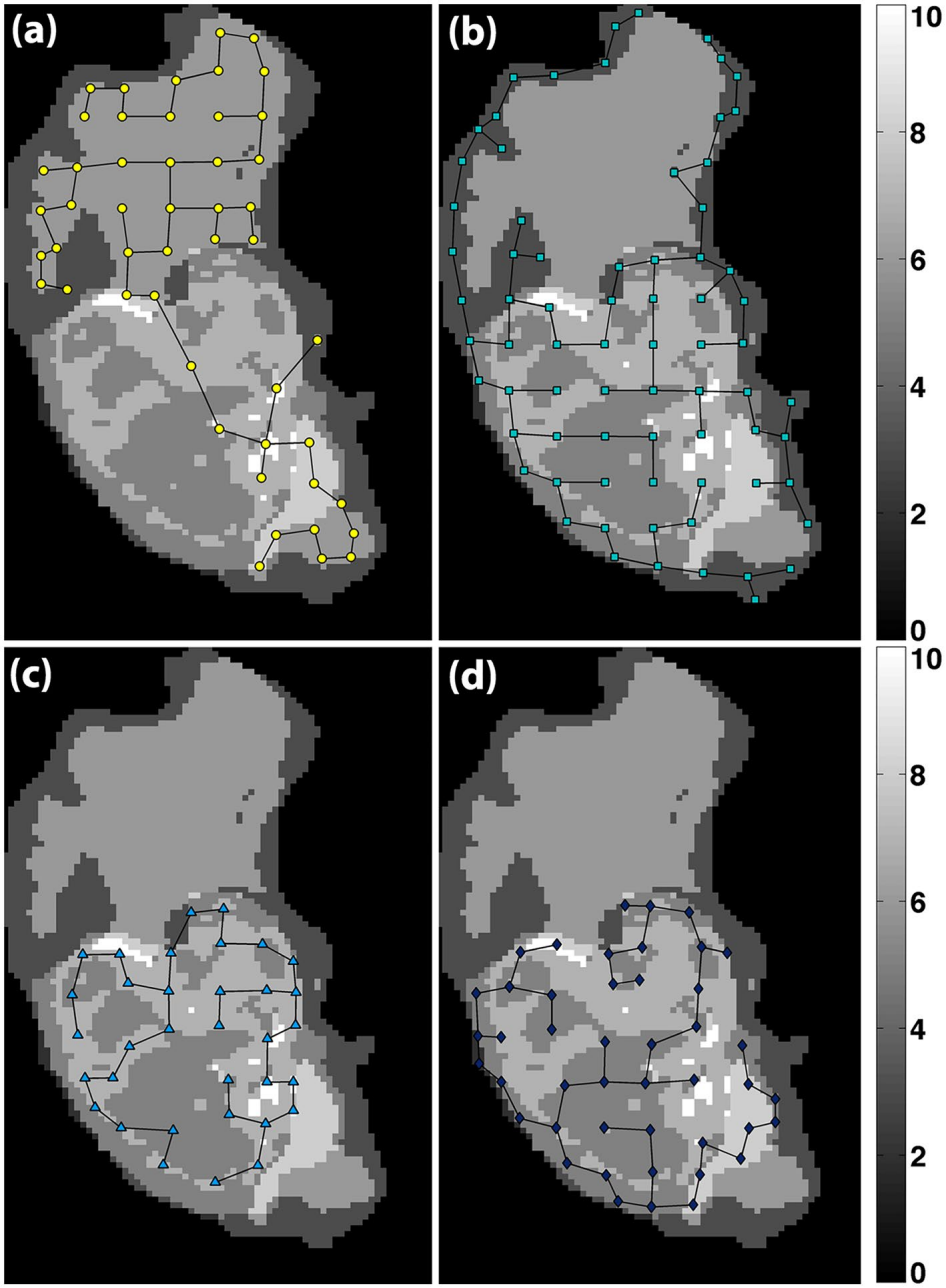
MSTs of the four habitats within the tumor ROI on an overlapped T1 and T2 ROI map. (**a**) T1 post-contrast high voxel intensity group, (**b**) T1 post-contrast low voxel intensity group, (**c**) T2 FLAIR high intensity voxel group, and (**d**) T2 FLAIR low intensity voxel group, respectively. Different gray levels within the ROI represent different habitats and their overlapping areas (scale 0–10).

We computed the coordinate of the centroid that specifies the center of mass of the region from each habitat inside of the small bounding grid box^32^. Coordinates from all grid boxes in each habitat were combined into one map. An MST was constructed across all these co-ordinates as vertices. Thus, we have four MSTs (one for each habitat: T1 high intensity group, T1 low, T2 high, or T2 low intensity group, respectively). Figure 2 shows MST of each of the four groups on an overlapped T1 and T2 ROI map. The mean, median, standard deviation, skewness, kurtosis, min/max ratio, and disorder of the branch lengths in MST were computed to obtain a set of seven features^18^ (for each habitat. Expressions for the features are listed below:

Mean edge weight, *f*_*µ*_:

1 $$ f_{\mu } = \frac{1}{n}\sum\limits_{i = 1}^{n} {w_{i} } $$

where *w*_*i*_ is an individual weight or branch length in MST.

Median edge weight, *f*_*median*_, is the value separating the higher half of branch lengths from the lower half.

Standard deviation of the distribution of edge weights, $f_{\sigma }$:

2 $$ f_{\sigma } = \left[ {\frac{1}{n}\sum\limits_{i = 1}^{n} {(w_{i} - f_{\mu } )^{2} } } \right]^{1/2} $$

Skewness of distribution of edge weights, *f*_*skewness*_:

3 $$ f_{skewness} = \frac{1}{{nf_{\sigma }^{3} }}\sum\limits_{i = 1}^{n} {\left( {w_{i} - f_{\mu } } \right)^{3} } $$

Kurtosis of distribution of edge weights, *f*_*kurtosis*_,:

4 $$ f_{kurtosis} = \frac{1}{{nf_{\sigma }^{4} }}\sum\limits_{i = 1}^{n} {\left( {w_{i} - f_{\mu } } \right)^{4} } $$

Finally, the min/max ratio, *f*_*r*_, is the ratio between maximum of **W** divided by the minimum value of **W** where **W** is a set of weights (branch lengths), ${\mathbf{W}} = \{ w_{1} ,w_{2} , \ldots ,w_{n} \}$.‘Disorder’ is the standard deviation $f_{\sigma }$ divided by the mean value of **W,** $f_{\mu }$**.**

### Feature extraction with graph run-length matrices (GRLM)

**Figure 3:**
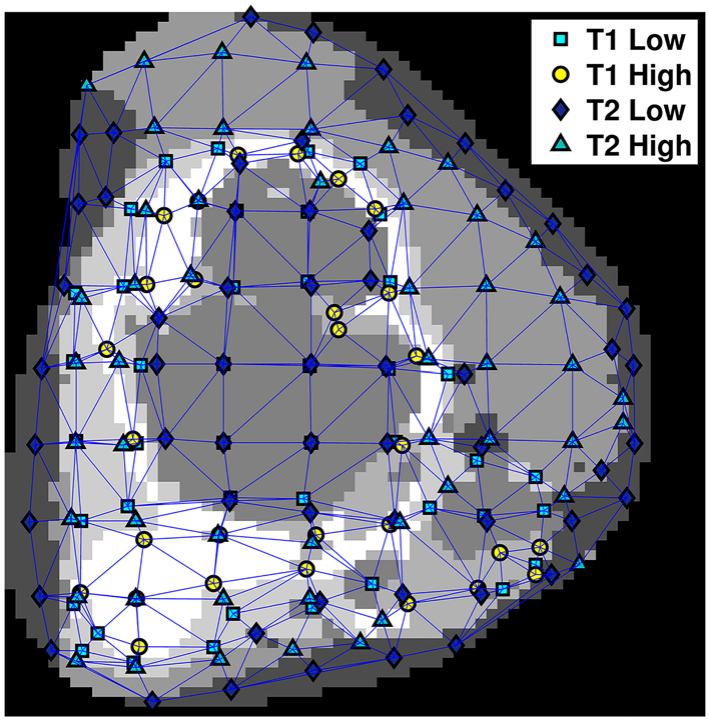
An example of ROI map with a Delaunay triangulation across all vertices. Different gray levels within the ROI represent different habitats and their overlapping areas.

Similar to the construction of the MST, we computed the coordinate of the centroid from each habitat inside of the rectangular grid box. For the extraction of GRLM features, we combined all coordinates from all four habitats into one set (map). Again, these four habitat groups represent T1 high- and low-intensity voxel groups and T2 high- and low-intensity voxel groups. Figure 3 shows an ROI map with all coordinates (across all four habitats) as vertices. Then, we constructed a Delaunay triangulation^33^ to connect these vertices. There are several ways to triangulate any given set of points and a Delaunay triangulation is one of the most widely used in scientific computing of various applications. In the Delaunay triangulation, all triangles for a set of points will have empty circumscribed circles^33^.

**Figure 4:**
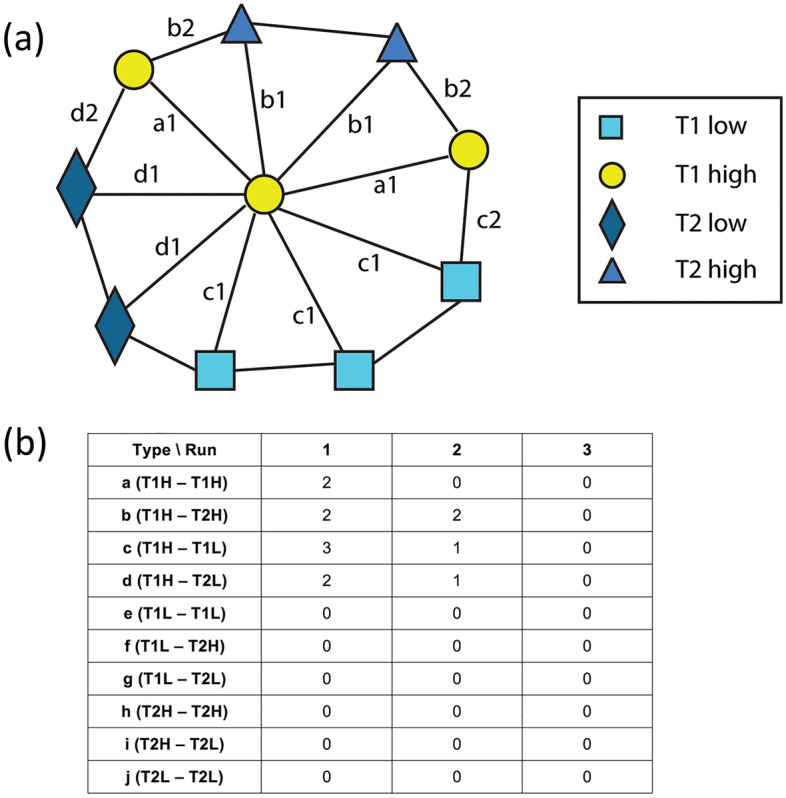
(**a**) Illustration of a single initial node located at the center and (**b**) a graph run-length matrix for this single initial node. This method was adapted from Tosun et al.

After constructing the ROI map with the Delaunay triangulation, a run-length matrix was computed. In this study, we used the GRLM method in the manner proposed by Tosun et al. for histopathological image segmentation^19^ because this aims to represent the spatial separation between point set entities. GRLM *G*(*t*, *l*) can be defined as the number of graph-edge runs with an edge type *t* and a path length *l* for a single node. The algorithm starts from the initial node to the furthermost node in the path within a circular window. Figure 4 shows the calculation of a graph run-length matrix for a single node. For the entire region, the algorithm accumulates the run-length matrices of the nodes in an ROI.

For extracting GRLM-based features, we used six measurements: short path emphasis (SPE), long path emphasis (LPE), edge type nonuniformity (ETN), and path length nonuniformity (PLN). The expressions for these features are listed below:

Short path emphasis (SPE) and SPE(*t*) for each edge type *t*:

5 $$ SPE = \frac{1}{{n_{r} }}\sum\limits_{t} {\sum\limits_{l} {G(t,l)/l^{2} } } $$

6 $$ SPE(t) = \frac{1}{{n_{r} (t)}}\sum\limits_{t} {\sum\limits_{l} {G(t,l)/l^{2} } } $$

where $n_{r}$ is the total number of runs in the GRLM and $n_{r} (t)$ is the total number of runs corresponding to edge type *t*.

Long path emphasis (LPE) and LPE(*t*) for each edge type *t*:

7 $$ LPE = \frac{1}{{n_{r} }}\sum\limits_{t} {\sum\limits_{l} {G(t,l) \times l^{2} } } $$

8 $$ LPE = \frac{1}{{n_{r} (t)}}\sum\limits_{t} {\sum\limits_{l} {G(t,l) \times l^{2} } } $$

Edge type nonuniformity (ETN) and path length nonuniformity (PLN):

9 $$ ETN = \frac{1}{{n_{r} }}\sum\limits_{t} {\left( {\sum\limits_{l} {G(t,l)} } \right)^{2} } $$

10 $$ PTN = \frac{1}{{n_{r} }}\sum\limits_{l} {\left( {\sum\limits_{t} {G(t,l)} } \right)^{2} } $$

### Classification of immune signature status

The immune signature scores from the TCGA cohort were dichotomized at the median value; either an up regulated as signature score > median value or down regulated as signature score ≤ median value. This binary designation is used as the class label in the classification task. These gene signatures associated with immune status in glioblastoma, immune effector response and immune suppression response, have been previously validated within glioblastoma and were evaluated in every patient in our dataset^21^.

### Statistical analysis

A total of 28 MST based features (seven MST-features from each of the four habitats) and 24 GLCM based (SPE, LPE, ETN, PTN, and 10 SPE(*t*) and 10 LPE(*t*)) features for 10 edge types were extracted and analyzed in this study. Survival was dichotomized at the 12 month time point (i.e. ≤ or > 12 months) based on the median overall survival duration, which is ranging from 9 to 15 months^1–3^ and imbalance in sample size of the two survival groups was controlled using class-proportional sampling^34^ in Waikato Environment for Knowledge Analysis (WEKA v3.7.12)^35^. Classification of the survival labels using all of the MST features was performed using random forest (RF) classification^36^ (10,000 trees using random forest classifier within WEKA). The important features were identified by using the Gini index, which calculates the amount of probability of a specific feature that is classified incorrectly when selected randomly.

In this study, we split the data into five subsets (5-folds) and rotating the training and validation among them using the fivefold cross-validation. Basically, cross-validation is one of the most powerful statistical techniques used to assess the performance of a predictive model by dividing the available dataset into subsets and using these subsets for both training and testing. The primary goal of cross-validation is to estimate how well a model will generalize to new, unseen data. The model performance was evaluated using the receiver operating characteristic (ROC) curve and the area under ROC curve (AUC). The ranking of the features was also obtained using a classifier-based attribute evaluator (where RF was set as the classifier) within WEKA. Sample size imbalance between classes is handled using class-proportional sampling^34^. The GRLM and MST features were computed in MATLAB (R2020a, The MathWorks, Inc.) using the formulas listed in this paper. The ROC, AUC, and classification using the RF classifier were computed using R (R Foundation for Statistical Computing, Vienna, Austria).

## Results

**Figure 5:**
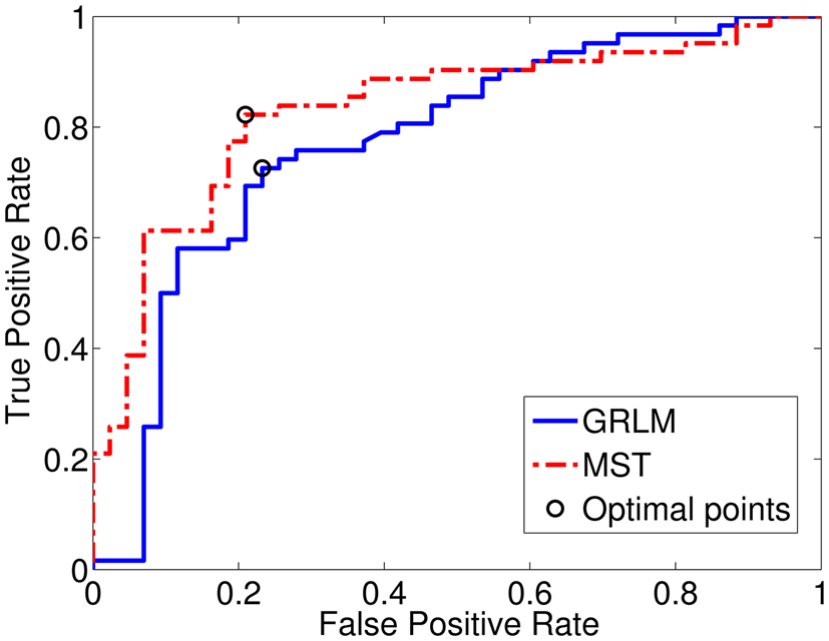
ROC curve for prediction of 12-month survival status. The x-axis is the false positive rate (or 1 – specificity); the y-axis is the true positive rate (or sensitivity). The area under the ROC curve is 0.832 for MST and 0.773 for GRLM. The optimal cut off points are (0.23, 0.73) and (0.21, 0.82) for GRLM and MST, respectively.

Classifier models were obtained using the random forest classification approach^36^ with fivefold cross-validation for the prediction of 12-month overall survival status based on all of the features derived from MST and GRLM-based methods, respectively. Figure 5 shows the ROC curves for the classifiers for MST and GRLM. The optimal cutoff point was determined by maximizing the sum of sensitivity and specificity. The results for the area under the ROC curves, the true positive rate, and true negative rate were summarized in Table 2. The area under the ROC curves was 0.832 for MST and 0.773 for GRLM. The accuracy was 80.7% for MST and 74.7% for GRLM computed using Eq. (11)

11 $$ ACC = \frac{TP + TN}{{TP + FN + TN + FP}} $$

where TP, FP, TN, and FN represent true positive, false positive, true negative, and false negative, respectively. The most important features based on rank (top five) are ratio of T1-low MST, ratio of T2-high MST, ratio of T1-high MST, disorder of T1-low MST, and standard deviation of T1-high for MST and LPE1, SPE4, LPE4, LPE10, and SPE10 for GRLM.

**Table 2:** The results of the ROC analysis for the 12-month survival prediction.

	AUC	MAE	TPR	TNR	ACC (%)
MST	0.832	0.384	0.823	0.791	80.7
GRLM	0.773	0.390	0.726	0.767	74.7

*AUC* area under ROC curve, *MAE* mean absolute error, *ACC* accuracy.

**Figure 6:**
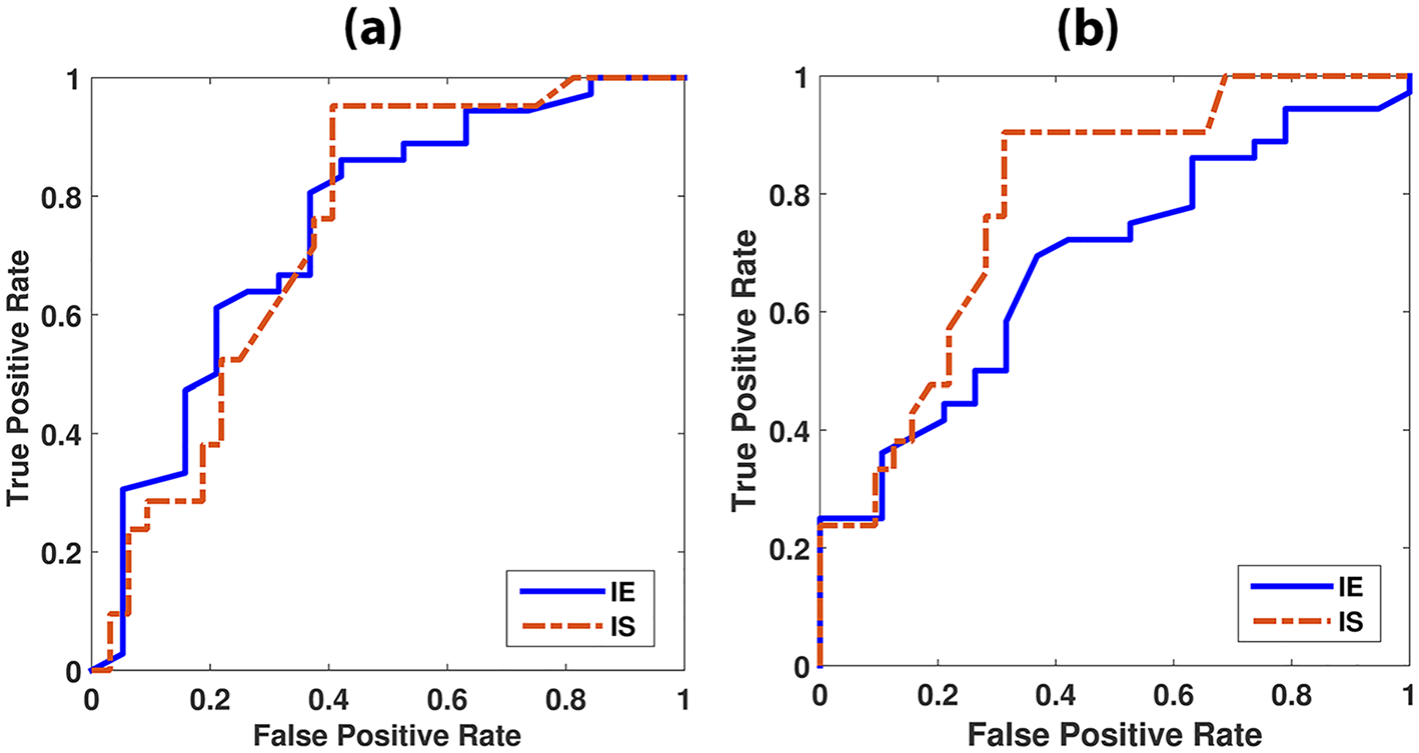
ROC curve for prediction of immune signature status for immune effector (IE) and immune suppressor (IS). The x-axis is the false positive rate (or 1 – specificity); the y-axis is the true positive rate (or sensitivity). (**a**) The area under the ROC curves (AUC) for MST are 0.747 and 0.743 for IE and IS, respectively, and (**b**) the area under the ROC curves for GRLM are 0.681 and 0.789, for IE and IS, respectively.

For classification of immune signature status, the accuracies from the random forest were 72.0% and 77.3% for IE and IS in MST, respectively, and 66.3% and 80.0% for IE and IS in GRLM, respectively. The AUC was 0.747 and 0.743 for IE and IS in MST, respectively, and 0.681 and 0.789 for IE and IS in GRLM, respectively. 95% confidence intervals (CI) of ROC are 93.7–96.4% for IS in MST and 88.5–92.3% for IS in GRLM, respectively. Table 3 summarized the true positive rate, true negative rate, classification accuracy, and 95% CI for MST and GRLM, and Fig. 6 shows the ROC curves for the classifiers for MST and GRLM, respectively. The optimal cutoff points were determined by maximizing the sum of sensitivity and specificity.

**Table 3:** The results of the ROC analysis for the immune status.

	Immune status	AUC	TPR	TNR	ACC (%)	95% CI (%)	
						Sensitivity	Specificity
MST	IE	0.747	0.861	0.579	72.0	83.8–88.2	54.8–61.0
	IS	0.743	0.952	0.594	77.3	93.7–96.4	56.3–62.5
GRLM	IE	0.681	0.694	0.632	66.3	66.4–72.3	60.1–66.2
	IS	0.789	0.905	0.688	80.0	88.5–92.3	65.8–71.7

## Discussion

In this study, we identified four distinct groups of voxel intensities (habitats) within the tumor ROI obtained from different MR sequences and separated them into high and low intensities using Gaussian mixture model. All studies were performed in a 2D slice with the maximum tumor area in both T1 post-contrast image and the T2 FLAIR image. These four habitats are considered as distinct entities within an ROI and their spatial relationships are characterized using MST and GRLM approaches. The MST is one of the most common characterization of the spatial proximity of points distributed topologically in space^37^. Gray-level run-length matrix is also a widely used method in texture analysis that characterizes image texture based on gray-levels run-length of image, introduced by Galloway^20^.

In this work, we applied these two methods to radiologically defined regions in glioblastoma tumors and investigated the association between graph-based features of habitat proximity from each method (MST or GRLM) with 12-month overall survival status in glioblastoma patients. In this study, we used random forest classification for the following reasons; (i) this classifier can handle large number of features, (ii) it works efficiently in scenarios where the number of instances is smaller than the number of features that are being used for classification, (iii) it is capable of performing cross-validation intrinsically, and (iv) it gives estimates of which variables are important in the classification^36^. Also, random forest models use several hundred decision trees (ensemble approach), each of them built over a random extraction of the observations from the dataset and a random extraction of the features, which mitigates the risk of overfitting and reduces the need for explicit feature selection.

The proposed MST and GRLM features with existing methods/features could assist towards the assessment of overall survival and serve as a prognostic tool based on routine MRI scans obtained in these patients. To our knowledge, this is the first instance of the investigation of both of these habitat proximity characterizations (MST- and GRLM-based analyses) in the context of multiparametric MRI data and for its application to survival prognostication in glioblastoma. Recently, researchers initiated standardizing the extraction of image biomarkers from acquired imaging for the purpose of high-throughput quantitative image analysis as radiomic features become popular and help quantifying characteristics present in medical image analysis. Although the GRLM and MST features were not included in the work, the image biomarker standardization initiative (IBSI), we adhere the guideline for the machine learning image analysis.

In this study, we used graph-based methods that could have potential advantages to connect between radiologic imaging and cellular evolution within tumors^31^. These results point to clinically relevant relationships of tumor-derived phenotype with overall survival. Such data could enable generation of valuable hypotheses for the investigation of phenotype relationships based on public domain datasets like TCGA. Further evaluation on clinically-matched patient cohorts with standardized imaging protocols is essential to strengthen evidence for the clinical translation of this finding. In this study, we also showed that these graph-based features are associated with immune signature status in glioblastoma patients with analysis of ROC curves, especially for immune suppressor in GRLM with an AUC value of 0.789.

Our study has a several limitations. First, one potential limitation is that this is a retrospective analysis performed using a publicly available database encompassing various scanning protocols and MRI systems resulting in differences in voxel resolution (256 × 256 or 512 × 512), slice thickness (1.4–5.0 mm), repetition time (4.9–3285.6 ms for T1 and 400–1100 ms for T2), and echo time (2.1–20 ms for T1 and 14–155 ms for T2). In this study, we performed image preprocessing steps such as voxel isotropic resampling and intensity normalization to make the MR image aspects comparable across various patients. However, these variations in MR images need to be examined more systematically with both MST- and GRLM-based features. Second, we focused on the slice with the maximum tumor area, which was due to a couple of factors such as balancing between accuracy, efficiency, and clinical relevance. As a preliminary study, we intend to pinpoint the region that potentially has the most significant impact on diagnosis and treatment decisions. However, the analysis with the entire tumor volume should be explored in the future study. Next, in this study, we performed a binary classification approach rather than a prediction of survival time. The reason for choosing the 12-month survival cutoff rather than utilizing a more continuous prediction of survival time is a matter that warrants clarification within the clinical context. Also, binary classification simplifies the outcome into distinct categories, which can be easier to interpret and communicate to both medical professionals and patients. However, using continuous prediction instead of binary classification in data analysis can offer several advantages. For example, continuous prediction provides a more detailed and nuanced understanding of outcomes. Also, continuous prediction retains the full range of survival times, preserving information that might be lost in binary categorization. Also, it can provide individualized predictions of survival time for each patient. This personalized information can guide treatment decisions, support patient counseling, and help tailor interventions to the unique circumstances of each individual. However, the choice should align with the research objectives and the utility of the outcomes in the given context. Since continuous prediction provides several advantages and valuable information, it needs to be examined in future studies. Lastly, for predicting immune signature status, we identified 34 patients based on the availability of information regarding immune signature status. Although we used cross-validation as the primary validation method, which is a valid and often preferred approach, especially when the dataset is limited in size, the future study should be explored with a larger dataset.

Glioblastoma is a highly aggressive and malignant type of brain tumor and researchers have been increasingly studying the differences in disease patterns and progression for brain cancers. Recently, several studies investigated sex-specific difference in progression and impact on survival for the glioblastoma patients^38,39^. They suggested that gaining a better understanding of the implications and interplay of sex differences in brain cancers will be informative for clinical practice and biological research, which could be considered in our future study with our proposed model.

In this study, we presented graph-based methods for characterizing the spatial proximity of radiologically-defined habitats in glioblastoma tumors, using a minimal spanning tree and graph run-length matrix construction. According to our results, the features from both MST- and GRLM-based methods provided quantitative metrics of image heterogeneity that have prognostic value for patient survival with high accuracy for both methods. We surmise that the spatial proximity features of habitats based on the MST and GRLM approaches may offer a promising method as a clinical prognostic tool in glioblastomas. However, this approach needs to be validated further in an independent patient cohort to confirm its predictive potential.

## Data Availability

The datasets generated during and/or analyzed during the current study are available from the corresponding author on reasonable request.
